# Supplementary material for: Wild cereal grain consumption among Early Holocene foragers of the Balkans predates the arrival of agriculture
Source: eLife. 2021 Dec 1;10:e72976. doi: 10.7554/eLife.72976 (PMC8782571; doi:10.7554/eLife.72976)
Supplement: Table 4—source data 1. [file elife-72976-table4-data1.docx]

| Species | Min. | Max. | Mean | Median | StDev. | Range | IQR |
| --- | --- | --- | --- | --- | --- | --- | --- |
| *A. caudata* | 5.29 | 59.3 | 21.6 | 16.7 | 15.17 | 5.29 – 59.33 | 26.55 |
| *A. comosa* | 7.95 | 34.5 | 21.5 | 21.7 | 9.78 | 7.95 – 34.54 | 20.09 |
| *A. crassa* | 13.38 | 53.7 | 35.3 | 33.7 | 11.09 | 13.38 – 53.69 | 19.08 |
| *A. cylindrica* | 8.52 | 54.0 | 24.2 | 23.7 | 13.07 | 8.52 – 54.05 | 21.6 |
| *A. geniculata* | 11.61 | 47.0 | 26.3 | 26.0 | 8.39 | 11.61 – 47.03 | 12.87 |
| *A. neglecta recta* | 10.54 | 62.7 | 35.0 | 36.2 | 14.46 | 10.54 – 62.71 | 26.5 |
| *A. peregrina* | 9.84 | 53.6 | 27.8 | 25.9 | 9.89 | 9.84 – 53.62 | 11.34 |
| *A. speltoides tauschii* | 13.25 | 40.0 | 23.5 | 22.2 | 5.93 | 13.25 – 39.97 | 8.39 |
| *A. triuncialis* | 5.60 | 50.1 | 28.2 | 28.2 | 11.24 | 5.60 – 50.06 | 15.18 |
| *A. uniaristata* | 14.35 | 62.4 | 38.2 | 39.3 | 12.87 | 14.35 – 62.38 | 22.83 |
| *A. ventricosa* | 14.10 | 40.0 | 26.3 | 25.7 | 7.44 | 14.10 – 40.04 | 12.77 |
| *H. vulgare distichon* | 5.19 | 29.6 | 19.7 | 22.2 | 8.12 | 5.19 – 29.59 | 8.32 |
| *T. dicoccum* | 6.17 | 41.5 | 16.5 | 12.8 | 8.66 | 6.17 – 41.55 | 14.07 |
| *T. monococcum* | 6.68 | 36.6 | 20.1 | 19.1 | 7.11 | 6.68 – 36.61 | 10.44 |
